# Utilizing portable electroencephalography to screen for pathology of Alzheimer’s disease: a methodological advancement in diagnosis of neurodegenerative diseases

**DOI:** 10.3389/fpsyt.2024.1392158

**Published:** 2024-05-24

**Authors:** Masahiro Hata, Yuki Miyazaki, Kohji Mori, Kenji Yoshiyama, Shoshin Akamine, Hideki Kanemoto, Shiho Gotoh, Hisaki Omori, Atsuya Hirashima, Yuto Satake, Takashi Suehiro, Shun Takahashi, Manabu Ikeda

**Affiliations:** ^1^ Department of Psychiatry, Osaka University Graduate School of Medicine, Osaka, Japan; ^2^ Department of Psychiatry, Esaka Hospital, Osaka, Japan; ^3^ Department of Occupational Therapy, Graduate School of Rehabilitation Science, Osaka Metropolitan University, Osaka, Japan; ^4^ Clinical Research and Education Center, Asakayama General Hospital, Osaka, Japan; ^5^ Department of Neuropsychiatry, Wakayama Medical University, Wakayama, Japan

**Keywords:** dementia, EEG, Alzheimer’s disease, amyloid beta, deep learning

## Abstract

**Background:**

The current biomarker-supported diagnosis of Alzheimer’s disease (AD) is hindered by invasiveness and cost issues. This study aimed to address these challenges by utilizing portable electroencephalography (EEG). We propose a novel, non-invasive, and cost-effective method for identifying AD, using a sample of patients with biomarker-verified AD, to facilitate early and accessible disease screening.

**Methods:**

This study included 35 patients with biomarker-verified AD, confirmed via cerebrospinal fluid sampling, and 35 age- and sex-balanced healthy volunteers (HVs). All participants underwent portable EEG recordings, focusing on 2-minute resting-state EEG epochs with closed eyes state. EEG recordings were transformed into scalogram images, which were analyzed using “vision Transformer(ViT),” a cutting-edge deep learning model, to differentiate patients from HVs.

**Results:**

The application of ViT to the scalogram images derived from portable EEG data demonstrated a significant capability to distinguish between patients with biomarker-verified AD and HVs. The method achieved an accuracy of 73%, with an area under the receiver operating characteristic curve of 0.80, indicating robust performance in identifying AD pathology using neurophysiological measures.

**Conclusions:**

Our findings highlight the potential of portable EEG combined with advanced deep learning techniques as a transformative tool for screening of biomarker-verified AD. This study not only contributes to the neurophysiological understanding of AD but also opens new avenues for the development of accessible and non-invasive diagnostic methods. The proposed approach paves the way for future clinical applications, offering a promising solution to the limitations of advanced diagnostic practices for dementia.

## 1 Background

Over 55 million people live with dementia globally, and the current annual cost associated with dementia is estimated at 1.3 trillion USD. Furthermore, the number of patients and related costs will continue to increase (1). Dementia has become a serious social and economic issue throughout the world, and it thus needs to be urgently addressed.

In 2021, the United States Food and Drug Administration (FDA) granted accelerated approval to aducanumab, a monoclonal antibody targeting amyloid beta (Aβ) aggregates, and this is the first approved drug to directly target the core pathophysiology of Alzheimer’s disease (AD). Thereafter, the FDA also granted traditional approval to lecanemab-irmb, the second medication targeting the fundamental pathophysiology of AD, in 2023. These approvals have initiated a new era in AD research, early biomarker-supported diagnosis, and biologically specific treatment (2).

A recent study using positron emission tomography (PET) showed a positivity rate for Aβ aggregates of only 63.8% in patients previously diagnosed with AD (3). Clinical AD diagnosis does not always rely on the presence of AD pathology (i.e., AD biomarker positivity) confirmed via cerebral spinal fluid (CSF) assays or PET; these should ideally be a prerequisite for beginning disease-modifying therapy (2). The identification of biomarkers can be invasive or costly and can only be performed in hospitals with state-of-the-art equipment (4). These limitations highlight the necessity of screening assessments that are widely available for screening in the rapidly increasing population of patients with dementia. One such tool for identifying biomarkers in clinical practice without these limitations is electroencephalography (EEG).

EEG signals derive from electromagnetic fields, stemming from the interactions of cortical neurons at a macroscopic scale (5). Consequently, EEG is regarded as a prime candidate for determining functional biomarkers for synaptic dysfunction and deterioration in dementia-related diseases (6). EEG is a noninvasive methodology, noted for its affordability, widespread availability, and sensitivity to the functional status of the brain (7). Recently, EEG has been utilized as a promising examination to screen for and assist in the diagnosis of dementia (8), yielding neurophysiological findings correlated with neurodegenerative diseases (7).

Regarding the association between EEG and AD biomarkers, patients with mild cognitive impairment (MCI) showed reduced alpha- and beta-frequency oscillations in CSF Aβ-positive cases compared with Aβ-negative cases (9). In addition, the same study showed that slowing of EEG activities were related to clinical progression in amyloid positive subjects with normal cognition (9). Another study indicated that abnormal neuronal activity, EEG-fMRI signal coupling, was associated with cerebral Aβ loads (10). In addition, we previously showed that theta EEG activities in patients with AD correlated with Aβ pathology traits determined using CSF analysis (11).

In this manner, findings on the relationships between neurophysiological features and AD biomarkers are accumulating. However, these studies were performed using clinical or research-quality EEG equipment, with many electrodes, and hold limitations in their applicability to the vastly increasing number of patients with dementia. In this study, we aimed to use a portable EEG system to identify patients with AD in a sample of patients with biomarker-verified AD, which could be useful for disease screening.

## 2 Methods

### 2.1 Study population

In this study, we recruited patients with probable AD. They visited the neuropsychological clinic in the psychiatry department of Osaka University Hospital. All patients underwent baseline assessments including demographic, cognitive, and neuropsychiatric assessments, brain structure assessments using magnetic resonance imaging (MRI) or computed tomography, laboratory measurements (e.g., blood cell count, blood chemistry measurements, thyroid hormones, vitamins B1, B12, and folic acid) between April 2021 and July 2023. Based on these examinations, we excluded patients with physical disorders that could affect cognition such as endocrine disorders, cerebral structural lesions, histories of brain injury, or drug/alcohol use disorders. All patients with suspected AD were diagnosed based using international standard criteria (12, 13). All diagnoses were performed by certified neuropsychiatrists through consensus at an expert conference in our department. In addition, diagnosis was confirmed using a CSF assay for the presence of the AD biomarker Aβ1–42/Aβ1–40 (14). This procedure is also used to select patients for treatment with lecanemab-irmb, the first approved disease-modifying drug for AD in Japan. In this study, we included patients who had undergone inpatient evaluations of cognitive function in our department and who underwent both CSF tests and portable EEG measurements as described below.

Age- and sex- balanced community-dwelling older persons were included as healthy volunteers (HVs). The inclusion criteria were: 1) no history of neurological or psychiatric diseases; 2) no history of severe head injury or alcohol/drug use disorder; and 3) no impairments in activities of daily living or global cognitive impairment (Mini-Mental State Examination [MMSE] (15) score ≥ 27) (16). MMSE is widely used as a screening test for cognitive dysfunction, and can evaluate, such as orientation, verbal memory, and general attention. All HVs underwent the same portable EEG measurements as the patient group.

Prior to enrollment, we explained the utilization of their clinical data for this research to all participants and obtained written informed consent. This study was approved by the ethical committee of Osaka University Hospital (approval number: 20312) and registered with the UMIN clinical trial registry (UMIN 000042903).

### 2.2 AD biomarkers in CSF samples

CSF samples were collected via lumbar puncture between 10:00 and 12:00 while patients were fasting. The first 1 mL of CSF obtained from each lumbar puncture was excluded from analysis. The samples were centrifuged at 430 × g for 5 minutes. The supernatant was aliquoted and stored at -80°C until analysis. Concentrations of Aβ1–40 and Aβ1–42 were measured in duplicate using ELISA kits (Human Aβ1–40 ELISA Kit Wako II (298–64601), Human Aβ1–42 ELISA Kit, High-Sensitive (296–64401); Fujifilm Wako Pure Chemical Corporation, Osaka, Japan) following the manufacturer’s instructions. We included patients with Aβ pathology traits determined by a CSF Aβ1–42/Aβ1–40 ratio lower than 0.0705 (17).

### 2.3 Portable EEG device

We used a multi-channel patch-type EEG system called “HARU-1,” which comprises a wireless sensing device and disposable electrode sheets. This device has received medical approval from Japan’s Pharmaceuticals and Medical Devices Agency, having been evaluated for its capability to measure EEG data using the same standards as traditional clinical EEG examinations (Certification Number: 302 AFBZX00079000, Class II, EEG; https://www.info.pmda.go.jp/ygo/pack/651319/302AFBZX00079000_1_01_02/302AFBZX00079000_1_01_02?view=body)(available on Feb 14, 2024). The electrode sheets of the device can be easily attached to the patient’s body, reducing discomfort on the skin of the forehead. The device is light at only 27 g and adopts a curved shape to fit the forehead. A wireless communication Bluetooth interface is used for device control. The device features a high voltage resolution of up to 24 bits (22 nV/LSB) and a low input-referred noise of 1 μVpp. It can record multi-channel EEG signals at a sampling rate of 250 Hz across three channels (center, left, right). The electrode sheets have a thickness of less than 50 μm, an elasticity of less than 200%, and a moisture permeability of 2700 g/m^2^/day. These sheets are manufactured using a cost-effective screen-printing process utilizing a biocompatible gel on an elastic base and silver-based material. The biocompatibility of conductive and non-conductive gels was assessed using ISO 10993 standards for skin sensitization, irritation, and *in vitro* cytotoxicity (18). Our previous analyzing data obtained with this portable EEG device demonstrated that characteristics of neuropsychiatric disorders could be precisely detected (19), indicating that this EEG system is robust enough for use in research. For photos of the device itself and people wearing it, please see the supplementary material in reference (19).

### 2.4 EEG measurements

We affixed the aforementioned EEG sheet to the study participants’ forehead. Then, we asked them to close their eyes and relax and began the EEG recording. When the EEG signals met quality-control criteria and the hum noise was less than 5 μV, the actual measurement was started. If the hum noise was found to be higher than this, the measurement was started after adjusting the noise to be less than 5 μV by moving the measuring sites. EEG measurements were conducted in environments other than shielded rooms, such as outpatient and inpatient rooms, after confirming that no noise was included as described above. EEG data from 2-minute resting-state epochs with closed eyes, monitored by the examinators, were analyzed in this study.

### 2.5 EEG signal processing and analysis

Based on the previous study that yielded promising results by converting EEG data to “scalograms” and identifying those data using “Vision Transformer (ViT)” (20), a deep learning model, we adopted a similar analysis for the EEG data in this study.

**Figure 1:**
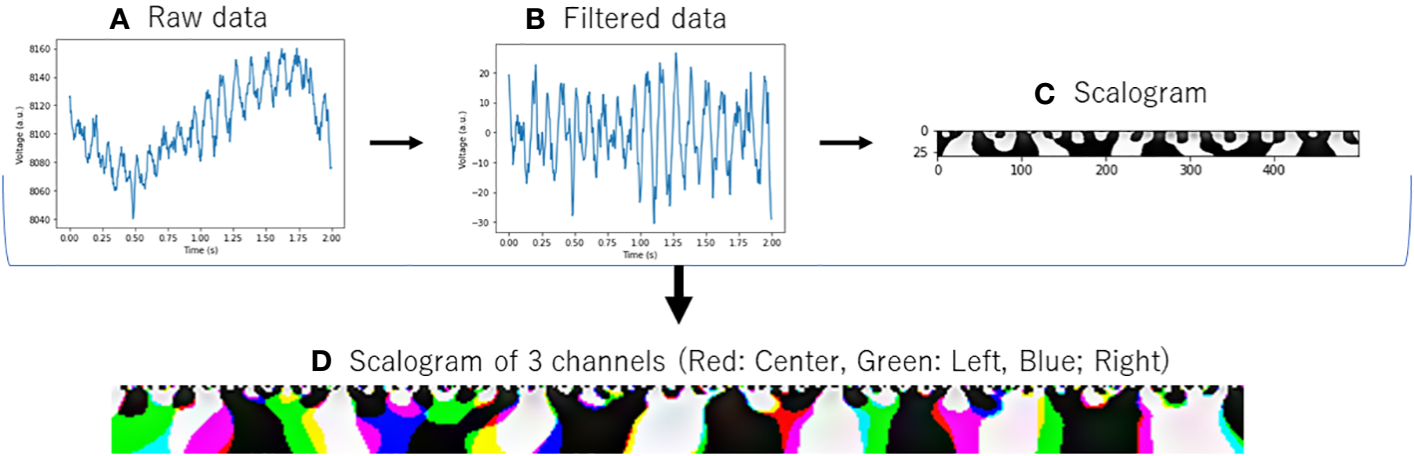
Conversion of EEG data to scalogram. **(A)** Raw data: Raw EEG data. **(B)** Filtered Data: EEG data with band-pass filter ranging from 4 to 75 Hz and a notch filter at 60 Hz. **(C)** Scalogram: Converted with Continuous Wavelet Transform from filtered data. **(D)** Scalogram of 3 channels: The same processing **(A–C)** was applied to data acquired from three distinct channels, and the resulting scalograms were color-coded and displayed in an integrated manner.

First, EEG data were converted to scalograms (**Figure 1**). The input data consisted of EEG signals with a sampling rate of 250 Hz across three channels. For each participant, segments of 120 seconds were recorded and subsequently segmented into 2-second epochs. As part of the data preprocessing procedure, a band-pass filter (4–75 Hz) and a notch filter (60 Hz) (Osaka, Japan, dealt with 60 Hz power line noise) were applied to attenuate power line noise and enhance the signal quality (21). The preprocessed data were subjected to continuous wavelet transform to convert them into scalograms (https://docs.scipy.org/doc/scipy/reference/generated/scipy.signal.cwt.html) (available on Feb 14, 2024). Then, the transformed scalograms of each channel were consolidated into a single composite colored image, with the center channel in red, the left channel in green, and the right channel in blue. This integration facilitated the preparation of the images for subsequent analysis using image-based deep learning models, ensuring efficient processing. Through this method, we could simultaneously analyze information from different channels, thereby gaining comprehensive insights.

**Figure 2:**
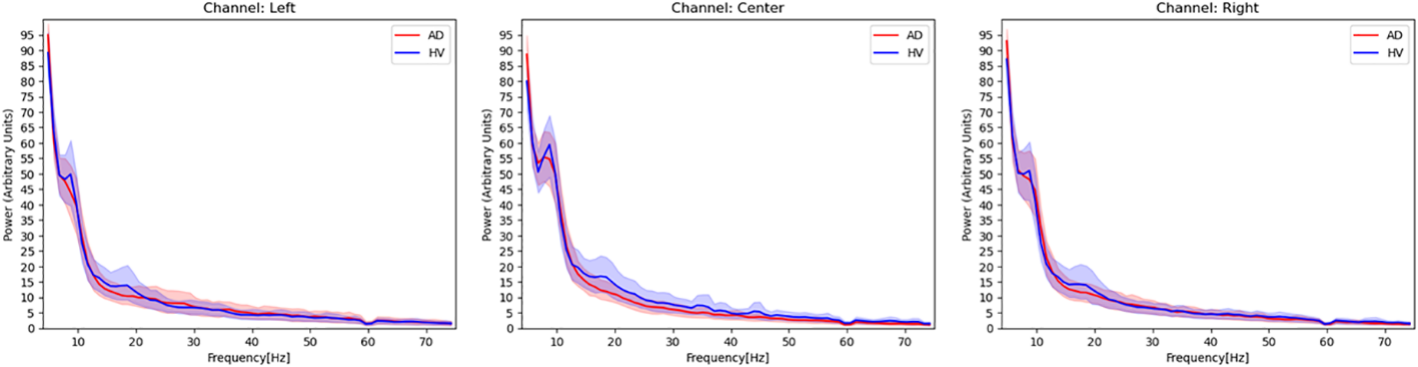
Power spectrum density estimated from EEG signals in patients with Alzheimer’s disease and healthy volunteers. Red activities in the figure represent the power spectrum density, with a 95% confidence interval, of patients with Alzheimer’s disease. Blue activities represent the power spectrum density of healthy volunteers at each EEG channel. The frequency range covered in this figure is 4–75 Hz, and a notch filter at 60 Hz has been applied.

Additionally, in order to grasp the overall trend of EEG activity of each channel, we examined the power spectrum density of the AD and HVs groups as in our previous study with this portable device (19) (**Figure 2**). The activities of both groups generally overlapped, with the absence of any biases or marked artifact. In this study, for the purpose of application to a simple screening test, the entire 2-minute resting eye-closure recordings of all subjects were used for analysis, without arbitrary artifact removal that would require specialized techniques, yielding no missing data. In our previous study (19) using the same portable EEG, we developed highly accurate prediction model without artifact rejection. Recent study that applied deep learning to EEG data had also reported that artifact rejection did not improve classification performance (22) and artifacts did not be excluded in the previous study (20) that aligned with this study.

Next, we used the deep learning model ViT. Recently, the transformer architecture has emerged as the predominant model in the field of natural language processing (23). This architecture enhances the efficiency of model training by enabling parallel processing of training data. However, the specific design of the transformer limits its direct applicability in computer vision. Addressing this limitation, one study (24) introduced the ViT model, a transformer variant tailored for computer vision tasks. According to experimental results, the ViT model not only demonstrated superior performance compared to leading convolutional neural network (CNN) models but also offered greater computational efficiency during training (25). Several articles have shown highly accurate identification results when applying ViT to EEG data (20, 26, 27). The ViT model is well-suited for effectively capturing the long-range correlations present in EEG signals. The self-attention mechanism of the ViT architecture allows it to model the complex spatio-temporal patterns in EEG data more accurately compared to conventional approaches like CNN, which are limited to learning local features. The feature of the ViT model to take holistic views of the input sequence holds an advantage for analyzing the intricate structure of EEG signals.

**Figure 3:**
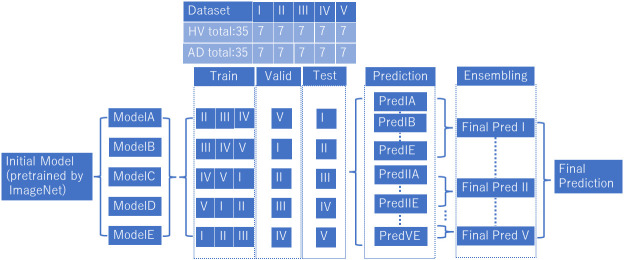
Vision transformer analytical model. We prepared model A to E, which learned wavelet-transformed images, and each trained model was used to discriminate between patients with AD and HVs by dividing the dataset into five subsets (I to V). We used an ensemble model after predictions (Predicted IA~VE) into final prediction (Final Pred I~V), and combined final predictions.

The ViT model used in this study was downloaded from https://github.com/huggingface/pytorch-image-models (available on Feb 14, 2024). The analytical model for ViT is shown in **Figure 3**. We prepared model A to E, which learned wavelet-transformed images, and each trained model was used to discriminate between patients with AD and HVs by dividing the dataset into five subsets (I to V) (i.e., 5-fold cross-validation). The ratio of training, validation, and test data was set at 3:1:1, with the prediction results from Model A using for test data 1 denoted as Pred I A. In order to train ViT, we did not add a shallow classifier and only performed fine tuning. Adam was employed as optimizer with a batch size set at 100. The training process was conducted over 5 epochs and early stopping was utilized to prevent overfitting. Through the process of ensembling, a majority vote was applied to the predictions from Pred I A to Pred I E. Furthermore, an average of 60 epochs (120 seconds) was calculated for each participant, with the majority result designated as Final Pred I. The aggregation of Final Pred I through Final Pred V was used to compute the final accuracy and area under receiver operating characteristic curve (AUC). This design ensured that the same participants were allocated to the same fold across all datasets, thereby preventing data leakage. These EEG analyses were performed using Python 3.8.13. (https://www.python.org/) (available on Feb 14, 2024).

### 2.6 Statistical analyses

For demographic data the Student’s t-test was used to compare ages patients with AD and HVs, the chi-squared test for sex, and the Mann–Whitney U test for MMSE scores. These statistical analyses were performed using SPSS software (v26; IBM Corp., Armonk, NY, USA). The threshold for significance was set at p = .05.

## 3 Results

### 3.1 Demographic data analysis

Demographic and clinical information for the both study groups are summarized in **Table 1**. We included 35 patients with biomarker-verified AD who underwent portable EEG recordings and same number of HVs during this study period. In comparisons between patient group and HVs, sex (p= .621) and age (p= .821) did not significantly differ; however, MMSE scores were significantly lower in the patient group (p<.001). The mean ratio of CSF Aβ1–42/Aβ1–40 in patients with biomarker-verified AD was 0.0483 ± 0.011.

**Table 1:** Demographic and clinical data of participants.

	AD	HVs	p-value
number of subjects	35	35	
gender(male/female)	12/23	14/21	0.621
age (years ± SD)	73.0 ± 9.6	72.5 ± 7.0	0.821
MMSE(scores ± SD)	19.5 ± 6.1	29.6 ± 0.7	<0.001

AD, patients with biomarker-verified Alzheimer’s disease.

HVs, Healthy Volunteers.

MMSE, Mini Mental State Examination.

### 3.2 Identifying patients with AD using EEG scalograms with ViT

**Figure 4:**
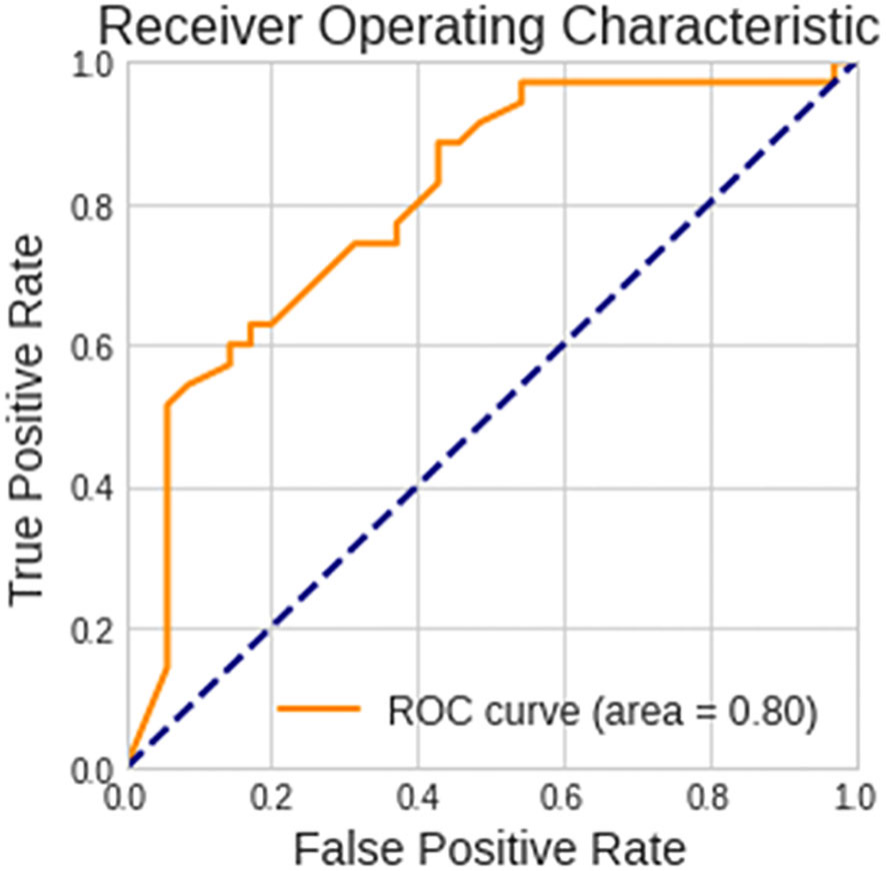
Performance of the vision transformer analytical model. The receiver operating characteristic curve for discerning between patients with biomarker-verified Alzheimer’s disease and healthy volunteers is shown.

Patients with biomarker-verified AD were identified with an accuracy of 73% and an AUC of 0.80, sensitivity of 88.6%, and specificity of 57.1%, based on images of EEG scalograms using the ViT analysis. The receiver operating characteristic curve is shown in **Figure 4**.

## 4 Discussion

This study employed a deep learning algorithm to identifying patients with biomarker-verified AD form EEG data measured using a patch-type portable device. To the best of our knowledge, this is the first study to explore the relationship between restricted-electrode EEG data and AD pathology. This study provides new insights into the neurophysiological features of AD pathology.

Several studies applying machine learning algorithms to EEG data features to identify patients with AD have been performed. Trambaiolli et al. (28) reported the best accuracy of 91% when combining several EEG parameters, especially alpha and beta activities, to discriminate patients with AD from age-matched HVs based on the feature selection algorithms. Another study investigated EEG spectral parameters for discriminating between patients with AD and age- and sex-matched HVs, yielding an accuracy of 0.67 and an AUC of 0.74 with corrected EEG features (29). A further EEG study applied time-frequency analysis with the Fourier and wavelet transforms and yielded an accuracy of 83% in the discrimination between patients with AD and HVs, providing superior performance in wavelet transforms used in this study (30). In studies using deep learning models, an applied convolutional neural network classifier showed a classification accuracy of 88% for patients with AD vs. HVs (31). In our previous study, a model leveraging deep learning techniques applied to EEG data demonstrated an accuracy of 82% in differentiating between HVs and patients with AD (32). However, these studies included cases of clinical AD diagnosis and lacked confirmed AD pathology. A rigorous and large-scale study reported a proportion of cases previously diagnosed with AD in which AD pathology was confirmed of 63.8% (3). Considering the prevalence of AD pathology, the accuracy of these studies might decrease by a certain degree, potentially aligning with the accuracy level of this study. In addition, these studies were conducted using 20-lead standard EEG, which is labor- and time-intensive; thus, the present study had an advantage in this respect.

There are also studies that have inferred AD pathology from other modalities, such as brain MRI. By utilizing image characteristics from brain MRI as input data and applying a machine learning model, patients with AD pathology were identified with an accuracy of 0.68 (cognitively normal) and 0.74 (subjects with MCI), with AUCs of 0.63 (cognitively normal) and 0.71 (subjects with MCI) using MRI characteristics and demographic data (33). In another study, the accuracy of predicting AD pathology using structural MRI data and machine learning was 0.697, and the AUC was 0.785 (34). While these discrimination accuracies are consistent with the findings of the current study, it should be noted that these results were obtained using brain MRI as input data. From the perspective of applicability in screening for AD pathology, the portable patch-type EEG system used here is more advantageous.

EEG measurements have been used clinically for excluding certain causes of dementia such as epilepsy; however, the system presented herein could lead to a new use for EEG measurements in screening for biomarker-verified AD. Recently, disease-modifying AD drugs have been approved in several countries; however, screening for suitability of such expensive drugs requires testing for Aβ using the expensive and restrictive PET or highly invasive spinal tap. Today, Aβ imaging technology is inaccessible to the vast majority of the world’s older adults who are at risk for dementia. We plan to further develop our deep-learning analysis for screening for suitability for treatment with disease-modifying AD drugs, taking advantage of this portable EEG device. This device does not require specific measurement locations such as shielded rooms and can be easily measured in a short time, so it is expected to be applied in a wide range of situations, such as medical checkups or nursing home.

The present results should be cautiously interpreted because of the study’s relatively small sample size. Additionally, this study included only cases who underwent inpatient evaluation and CSF testing, which might have yielded a selection bias. Future validation using larger outpatient and inpatient samples at multiple institutions are required to enlarge the generalizability of these results. The HVs in this study were not tested for AD biomarkers due to the invasiveness of the sampling method; the possibility cannot be ruled out that some AD biomarker-positive cases were inadvertently included in the control group.

## 5 Conclusions

In this study, we developed a system using a patch-type portable EEG device and an advanced deep learning algorithm specifically designed to discern AD pathology. Utilizing the mobility offered by portable EEG technology, we aim to employ this newly developed system as a foundational tool for the screening of biomarker-verified AD in diagnostic practice. This approach not only represents a significant methodological innovation but also holds the potential to substantially enhance the efficacy and accessibility of AD screening protocols.

## Data availability statement

The raw data supporting the conclusions of this article will be made available by the authors, without undue reservation.

## Ethics statement

The studies involving humans were approved by the ethical committee of Osaka University Hospital. The studies were conducted in accordance with the local legislation and institutional requirements. The participants provided their written informed consent to participate in this study.

## Author contributions

MH: Conceptualization, Data curation, Formal analysis, Funding acquisition, Investigation, Methodology, Project administration, Resources, Software, Supervision, Validation, Visualization, Writing – original draft, Writing – review & editing. YM: Conceptualization, Data curation, Formal analysis, Funding acquisition, Investigation, Methodology, Project administration, Resources, Software, Supervision, Validation, Visualization, Writing – original draft, Writing – review & editing. KM: Data curation, Methodology, Supervision, Writing – review & editing. KY: Data curation, Methodology, Supervision, Writing – review & editing. SA: Data curation, Methodology, Supervision, Writing – review & editing. HK: Data curation, Methodology, Supervision, Writing – review & editing. SG: Data curation, Methodology, Supervision, Writing – review & editing. HO: Data curation, Methodology, Supervision, Writing – review & editing. AH: Data curation, Methodology, Supervision, Writing – review & editing. YS: Data curation, Methodology, Supervision, Writing – review & editing. TS: Data curation, Methodology, Supervision, Writing – review & editing. ST: Data curation, Methodology, Supervision, Writing – review & editing. MI: Data curation, Methodology, Supervision, Writing – review & editing.
